# Prognostic Implication of a Novel Metabolism-Related Gene Signature in Hepatocellular Carcinoma

**DOI:** 10.3389/fonc.2021.666199

**Published:** 2021-06-04

**Authors:** Chaoyan Yuan, Mengqin Yuan, Mingqian Chen, Jinhua Ouyang, Wei Tan, Fangfang Dai, Dongyong Yang, Shiyi Liu, Yajing Zheng, Chenliang Zhou, Yanxiang Cheng

**Affiliations:** ^1^ Department of Gynecology, Minda Hospital of Hubei Minzu University, Enshi, China; ^2^ Department of Obstetrics and Gynecology, Renmin Hospital of Wuhan University, Wuhan, China; ^3^ Department of Intensive Care Unit, Renmin Hospital of Wuhan University, Wuhan, China

**Keywords:** hepatocellular carcinoma, metabolism-related genes, prognostic signature, overall survival, immunotherapy

## Abstract

**Background:**

Hepatocellular carcinoma (HCC) is one of the main causes of cancer-associated deaths globally, accounts for 90% of primary liver cancers. However, further studies are needed to confirm the metabolism-related gene signature related to the prognosis of patients with HCC.

**Methods:**

Using the “limma” R package and univariate Cox analysis, combined with LASSO regression analysis, a metabolism-related gene signature was established. The relationship between the gene signature and overall survival (OS) of HCC patients was analyzed. RT-qPCR was used to evaluate the expression of metabolism-related genes in clinical samples. GSEA and ssGSEA algorithms were used to evaluate differences in metabolism and immune status, respectively. Simultaneously, data downloaded from ICGC were used as an external verification set.

**Results:**

From a total of 1,382 metabolism-related genes, a novel six-gene signature (G6PD, AKR1B15, HMMR, CSPG5, ELOVL3, FABP6) was constructed based on data from TCGA. Patients were divided into two risk groups based on risk scores calculated for these six genes. Survival analysis showed a significant correlation between high-risk patients and poor prognosis. ROC analysis demonstrated that the gene signature had good predictive capability, and the mRNA expression levels of the six genes were upregulated in HCC tissues than those in adjacent normal liver tissues. Independent prognosis analysis confirmed that the risk score and tumor grade were independent risk factors for HCC. Furthermore, a nomogram of the risk score combined with tumor stage was constructed. The calibration graph results demonstrated that the OS probability predicted by the nomogram had almost no deviation from the actual OS probability, especially for 3-year OS. Both the C-index and DCA curve indicated that the nomogram provides higher reliability than the tumor stage and risk scores. Moreover, the metabolic and immune infiltration statuses of the two risk groups were significantly different. In the high-risk group, the expression levels of immune checkpoints, TGF-β, and C-ECM genes, whose functions are related to immune escape and immunotherapy failure, were also upregulated.

**Conclusions:**

In summary, we developed a novel metabolism-related gene signature to provide more powerful prognostic evaluation information with potential ability to predict the immunotherapy efficiency and guide early treatment for HCC.

## Introduction

As the sixth most common cancer worldwide, liver cancer has become the fourth most prevalent cause of tumor-associated deaths globally (1). Hepatocellular carcinoma (HCC) is considered the most common pathological type of primary malignant liver cancer and has become a significant global health concern (2). At the time of diagnosis, the vast majority of HCC patients are in the middle-to-late stages of the disease. Although surgery and multi-mode treatment have been improved, the overall 5-year survival rate of HCC patients remains low, approximately 10–20%, due to its invasiveness, metastatic potential, and recurrence rate (3). Therefore, identifying effective biomarkers for HCC prognosis is important for the evaluation and treatment of HCC. Common prognostic indicators of HCC include clinicopathological features, such as alpha-fetoprotein and microvessel infiltration (4, 5). However, the specificity and sensitivity of existing prognostic markers do not provide meaningful prognosis patterns.

Recent studies have indicated that metabolic changes associated with cancer represent a novel concern in managing this disease. Many studies have confirmed that changes in cell metabolism play a critical role in the initiation and progression of cancer (6–8). The liver is an important metabolic organ, playing a critical role in many important metabolic pathways including glycolysis, the tricarboxylic acid cycle, and nucleotide biosynthesis (9). Therefore, elucidating the relationship between metabolism-related genes and HCC is essential for understanding its pathogenesis (10). However, the value of a metabolism-related gene signature in HCC prognosis evaluation remains unclear.

In this study, we first downloaded transcriptome profiling data and the corresponding clinical information of liver hepatocellular carcinoma (LIHC) from The Cancer Genome Atlas (TCGA) and International Cancer Genome Consortium (ICGC) databases, respectively. We then used the least absolute contraction and selection operator (LASSO) regression analysis to construct a six-gene signature related to metabolism in the TCGA set, and verified it in the ICGC set. Using the six-gene signature, we accurately predicted the overall survival (OS) of patients with HCC. Finally, we identified six differentially expressed genes (DEGs) with prognostic value to construct a signature, which represents a promising predictive indicator for HCC patients.

## Materials and Methods

### Data Collection

We downloaded transcriptome profiling data (fragments per kilobase million, FPKM), up to and including October 20, 2020, of 374 HCC patients and 50 normal tissues, along with the corresponding clinical information of 377 patients with HCC from the TCGA database (https://portal.gdc.cancer.gov/repository), as a training set. The clinical information included survival time, survival status, age, sex, histological grade, tumor stage, and TNM stage. Clinical characteristics are shown in **Table 1**. Subsequently, these data were matched according to the sample names, and the date of 343 HCC patients who survived more than 30 days was used for follow-up analyses to rule out non-neoplastic causes of death, including heart failure, infection, and bleeding. We then evaluated the other 239 HCC samples by using complete expression profiling data downloaded from the ICGC database (https://dcc.icgc.org/) as a validation set. These samples included liver cancer tissues from Japan with a background of hepatitis B virus (HBV) and hepatitis C virus (HCV) infection. The clinical information of 259 HCC patients, including survival time, survival status, age, sex, and tumor stage of the corresponding patients, was also obtained and extracted (**Table 2**). After matching these data according to sample names, 231 patients with HCC were evaluated. Homo sapiens GTF files in the Ensembl database were used to annotate the probes.

**Table 1:** Clinical characteristics of the HCC patients in the TCGA database.

Covariates	Type	Total (n = 349)
Age (%)	<65	208 (59.60)
	≥65	141 (40.40)
Gender (%)	Male	238 (68.19)
	Female	111 (31.81)
Vital status (%)	Alive	224 (64.18)
	Dead	125 (35.82)
Tumor grade (%)	I	53 (15.19)
	II	164 (46.99)
	III	114 (32.66)
	IV	13 (3.73)
	Unknown	5 (1.43)
Tumor Stage (%)	I	165 (47.28)
	II	78 (22.35)
	III	81 (23.21)
	IV	3 (0.86)
	Unknown	22 (6.30)
T Stage (%)	T1	172 (49.28)
	T2	85 (24.36)
	T3	76 (21.78)
	T4	13 (3.72)
	Unknown	3 (0.86)
M Stage (%)	M0	251 (71.92)
	M1	3 (0.86)
	Unknown	95 (27.22)
N Stage (%)	N0	244 (69.91)
	N1	3 (0.86)
	Unknown	102 (29.23)

**Table 2:** Clinical characteristics of the HCC patients in the ICGC database.

Covariates	Type	Total (n = 258)
Age (%)	<65	91 (35.27%)
	≥65	167 (64.73%)
Gender (%)	Male	190 (73.64%)
	Female	68 (26.36%)
Vital status (%)	Alive	214 (82.95%)
	Dead	44 (17.05%)
Tumor Stage (%)	I	40 (15.50%)
	II	117 (45.35%)
	III	78 (30.23%)
	IV	23 (8.92%)

### Establishment of a Prognostic Gene Signature That Related to Metabolism

Metabolism-related genes were collected from the Molecular Signatures Database (MSigDB) (REACTOME_METABOLISM_OF_AMINO_ACIDS_AND_DERIVAT IVES M727, REACTOME_METABOLISM_OF_CARBOHYDRATES M16864, and REACTOME_METABOLISM_OF_LIPIDS M27451). The “Limma” R package was used to screen metabolism-related DEGs between tumor and paired adjacent non-tumor tissues (FDR <0.05, |log_2_FC| >3). We then applied univariate Cox analysis to identify the prognostic metabolism-related DEGs. Based on these overlapping prognostic DEGs, an interactive network was generated using the STRING dataset. To avoid overfitting, the “glmnet” R software package was used for LASSO analysis to construct a prognostic signature. The following formula was applied to the definition of the risk score for each patient (11):

Risk score=βgene(1)×E gene(1)+βgene(2)× E gene(2)+…+βgene(n)×E gene(n)

E denotes the normalized expression level of the gene, and β denotes the corresponding regression coefficient. Patients obtained from the TCGA and ICGC databases were divided into two risk groups respectively, according to the median risk score of the TCGA dataset.

### Verification of the Prognostic Evaluation Efficiency of the Gene Signature

To identify the correlations between gene signature and the clinical outcomes of HCC patients, we conducted Kaplan–Meier survival analysis to assess the relationship between risk grouping and OS. The “survivalROC” R package was utilized to execute receiver operating characteristic (ROC) analysis to estimate the specificity and sensitivity of prognostic evaluation of gene signature prognosis assessment. Principal components analysis (PCA) and t-distributed stochastic neighbor embedding (t-SNE) were used to investigate the distribution patterns of the two groups. Moreover, we conducted an independent prognostic analysis to evaluate the independent risk predictors for the prognosis of HCC patients.

### Verification of the mRNA and Protein Expression of Six Metabolism-Related Genes Between HCC and Adjacent Normal Liver Tissues

Three paired HCC samples and adjacent normal liver tissue samples were collected. Total RNA was extracted from the tissues by using RNAiso Plus (Takara, Otsu, Japan) according to the manufacturer’s instructions. Next, we reverse-transcribed RNA into cDNA by HiScript^®^ III RT SuperMix for qPCR Kit (Vazyme). The ChamQ SYBR qPCR Master Mix Kit (Vazyme) was used to conduct real-time reverse transcription polymerase chain reaction (RT-PCR) using Bio‐Rad CFX96™ (Bio‐Rad, Hercules, California, USA). Gene expression was standardized as the beta-actin gene ACTB. The sequences of the primers for the prognostic genes and ACTB are presented in **Table S1**. Each sample was repeated three times. The 2^−ΔΔCt^ method was used to compare the relative expression levels of metabolism-related genes in HCC and adjacent normal liver tissues. Representative immunohistochemical staining images of the six metabolism-related genes in HCC and adjacent normal liver tissues were downloaded from the Human Protein Atlas (HPA) database (https://www.proteinatlas.org/).

### Construction of Nomogram

Nomogram analysis was carried out with the “rms” R package, by fitting the predictors significantly related with OS of HCC in multivariate analysis into research to predict the OS of patients with HCC. A calibration plot was constructed to evaluate the discrimination between the 1-, 2-, and 3-year OS predicted by the nomogram, and the actual values. Furthermore, the reliability of the nomogram was estimated using the concordance index (C-index) and decision curve analysis (DCA).

### Functional Enrichment Analysis

We further conducted a gene set enrichment analysis (GSEA) for functional annotation. Moreover, single-sample gene set enrichment analysis (ssGSEA) in the “GSVA” R package was performed to quantify the difference in tumor-infiltrating immune cell scores and immune-related pathway activity between the two risk groups.

### Statistical Analysis

R software and GraphPad Prism 7 were used for all statistical analyses. Differences between the two groups were assessed using the Student’s t-test. Statistical significance was set at *P <*0.05.

## Results

### Acquisition of Prognostic Metabolism-Related DEGs

**Scheme 1:**
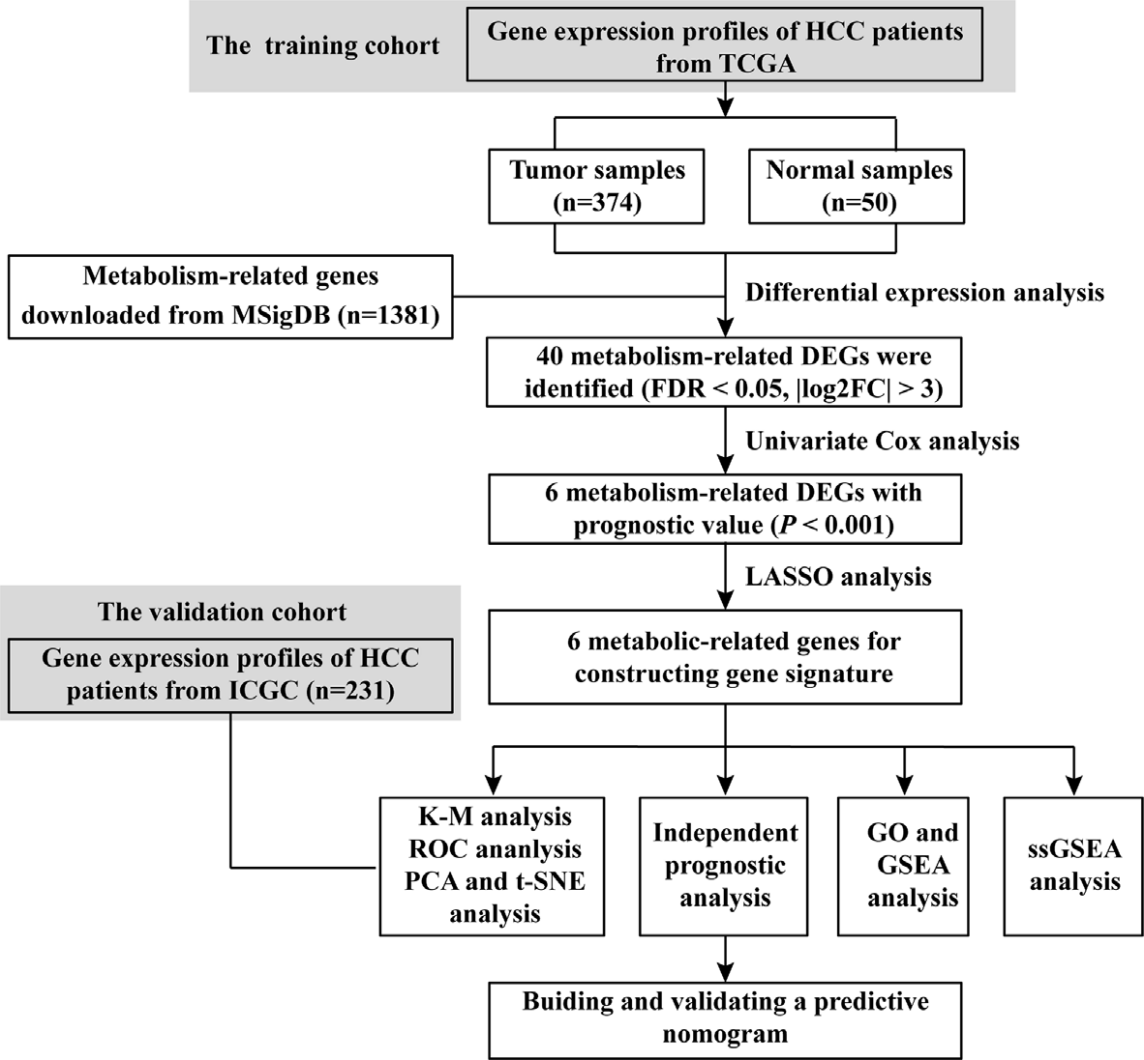
Flow chart of data collection and analysis.

**Figure 1:**
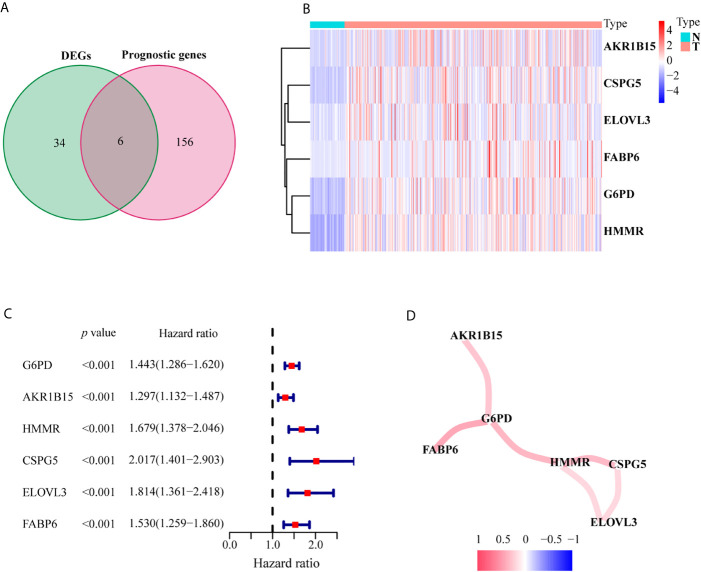
Identification of the candidate metabolism-related genes in the TCGA set. **(A)** Venn diagram to identify prognostic DEGs between tumor and adjacent normal tissue. **(B)** The heatmap of the six genes between tumor and adjacent normal tissue. **(C)** The forest plot between gene expression and OS. **(D)** The correlation network of six metabolism-related genes.

A flowchart of this process is displayed in **Scheme 1**. A total of 1,382 metabolism-related genes were collected from the MSigDB (**Table S2**). Among them, 40 metabolism-related genes were differentially expressed between HCC and adjacent paired normal tissues (FDR <0.05, |log_2_FC| >3, **Table S3**). Meanwhile, 162 genes related to HCC prognosis were screened out using univariate Cox analysis (*P <*0.001, **Table S4**). Subsequently, six prognostic DEGs (G6PD, AKR1B15, HMMR, CSPG5, ELOVL3 and FABP6) were identified for further analysis (**Figure 1A**). **Figures 1B, C** present the heatmap and forest plots of the six prognostic DEGs. All six genes were defined as deleterious effectors with all-hazard ratio (HR) values >1. The correlation network between the six metabolism-related genes is shown in **Figure 1D**.

### Establishment of a Prognostic Metabolism-Related Gene Signature

**Figure 2:**
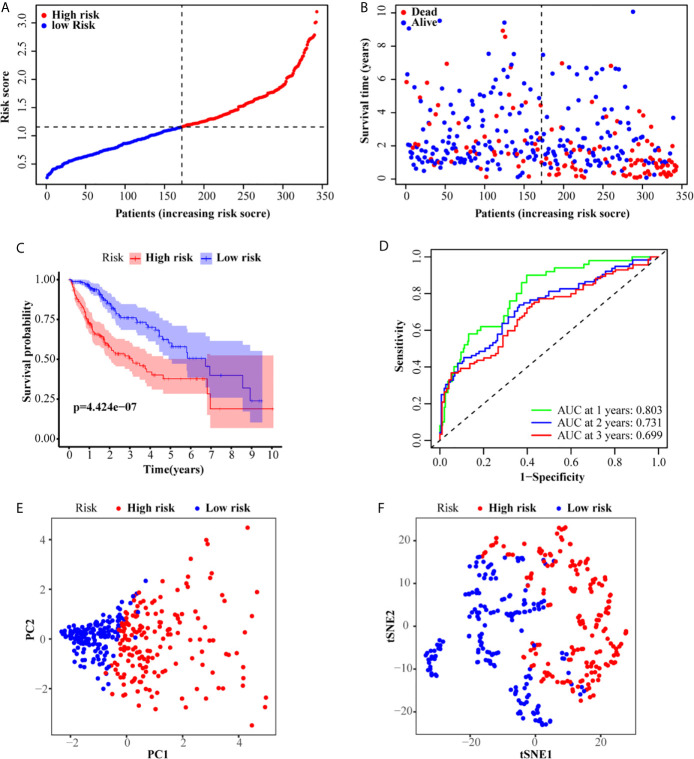
Prognostic analysis of the six-gene signature in the TCGA set. **(A, B)** The distribution and median value of the risk scores in the TCGA set. **(C)** Kaplan–Meier curves for the OS of patients in the high-risk group and low-risk group. **(D)** AUC of time-dependent ROC curves in the TCGA set. **(E)** PCA and **(F)** t-SNE analysis of the ICGC set.

LASSO analysis was performed to identify a six-gene signature according to the optimal value of λ. The risk score of patients was determined as follows: Risk score = 0.209817253236067 × E (G6PD) + 0.123704944063381 × E (AKR1B15) + 0.230463210311424 × E (HMMR) + 0.21119779243939 ×E (CSPG5) + 0.271858044998993 × E (ELOVL3) + 0.0373144717068065 × E (FABP6). According to the median risk scores, 343 HCC patients were divided into a high-risk group (171 patients) and a low-risk group (172 patients). The clinical characteristics of the patients are described in **Table 3**. As depicted in **Figures 2A, B**, an increase in risk score was related to poor OS. The Kaplan–Meier curve analysis showed that patients in the high-risk group exhibited reduced OS (*P <*0.001, **Figure 2C**). Time-dependent ROC analysis was used to evaluate the prognostic evaluation ability of a six-gene signature (**Figure 2D**). The area under the curve (AUC) at 1-, 2-, and 3-year OS were 0.803, 0.731, and 0.699, respectively. Moreover, PCA and t-SNE analyses were used to identify the different distributions between the two risk groups. As shown in **Figures 2E, F**, the distribution patterns of patients in the two groups were different.

**Table 3:** Baseline characteristics of the patients in different risk groups.

Characteristics	TCGA cohort			ICGC cohort		
	High risk	Low risk	*P* value	High risk	Low risk	*P* value
**Age**			0.8604			0.0223
<65	103	102		55	27	
≥65	68	70		120	29	
**Gender**			<0.0001			0.4411
Female	52	58		44	17	
Male	119	114		131	39	
**Vital status**			<0.0001			0.0960
Alive	92	128		139	50	
Dead	79	44		36	6	
**Grade**			0.0152*			
I/II	95	119				
III/IV	74	50				
Unknown	2	3				
**Stage**			0.1273			0.3749
I/II	110	128		104	37	
III/IV	48	35		71	19	
Unknown	13	9		0	0	
**T stage**			0.0402*			
T1/T2	120	132				
T3/T4	51	37				
Unknown	0	3				
**M stage**			0.8220*			
M0	124	121				
M1	1	2				
Unknown	46	49				
**N stage**			1.0000*			
N0	119	120				
N1	2	1				
Unknown	50	51				

*Fisher’s test.

### Verification of the Metabolism-Related Gene Signature

**Figure 3:**
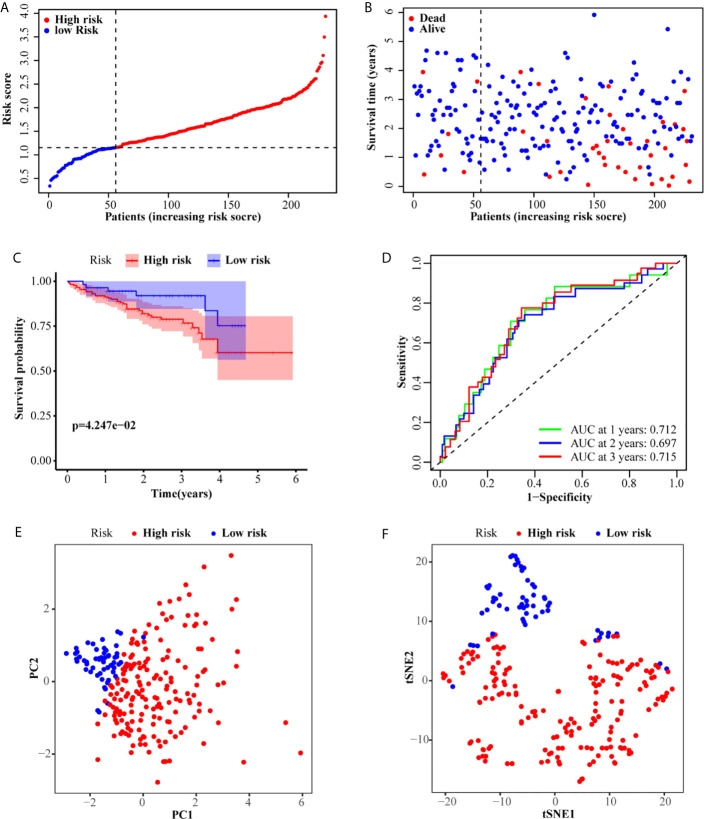
Validation of the six-gene signature in the ICGC set. **(A, B)** The distribution and median value of the risk scores in the ICGC set. **(C)** Kaplan–Meier curves for the OS of patients in the high-risk group and low-risk group. **(D)** AUC of time-dependent ROC curves in the ICGC set. **(E)** PCA and **(F)** t-SNE analysis of the ICGC set.

According to the median risk score of the TCGA set, 231 patients from the ICGC set were divided into a high-risk group (175 patients) and a low-risk group (56 patients). Similar to the TCGA set, patients in the low-risk group exhibited better OS (**Figures 3A, B**). The Kaplan–Meier curve also confirmed that the OS of patients in the high-risk group was worse than that of patients in the low-risk group (*P <*0.001, **Figure 3C**). Similarly, the AUC of the six-gene signature was 0.712, 0.697, and 0.715 at the 1-, 2-, and 3-year timepoints, respectively (**Figure 3D**). Additionally, PCA and t-SNE also showed that the two groups were presented in two different patterns (**Figures 3E, F**).

### Performance Comparison of the Metabolism-Related Gene Signature With Other Gene Signatures in Prognosis Evaluation

**Figure 4:**
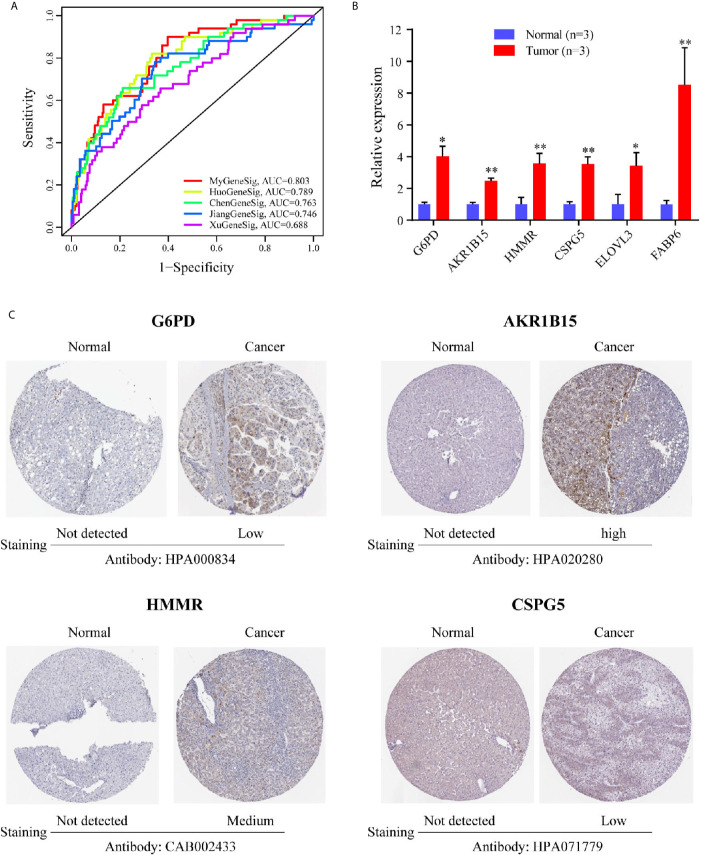
The expression level of six genes in HCC. **(A)** The ROC analysis at 1 year of overall survival for the MyGeneSig, HuoGeneSig, ChenGeneSig, JiangGeneSig, and XuGeneSig. **(B)** The mRNA expression analysis by qRT-RCR. **(C)** The immunohistochemistry staining images of G6PD, AKR1B15, HMMR and CSPG5 from the HPA. *P<0.05; **P<0.01.

We further compared the prediction performance of the metabolism-related gene signature with four other published gene signatures obtained from Huo’s (12), Chen’s (13), Jiang’s (14) and Xu’s (15) studies, in the TCGA database. **Figure 4A** reveals that the AUC of the metabolism-related gene signature for 1-year OS was 0.803, which is significantly larger than that of Huo’s (0.789), Chen’s (0.769), Jiang’s (0.746) and Xu’s (0.688) gene signatures. The results demonstrate that the metabolism-related gene signature offers superior prognosis evaluation performance than the four previously published gene signatures for HCC.

### Verification of the Expression of Six Metabolism-Related Genes Between HCC and Adjacent Normal Liver Tissues

To evaluate the expression of the six metabolism-related genes (G6PD, AKR1B15, HMMR, CSPG5, ELOVL3, and FABP6) between HCC and adjacent normal liver tissues, qRT-PCR was performed to quantify mRNA expression levels. The results of qRT-PCR revealed that in comparison with adjacent normal liver tissues, the six metabolism-related genes were all upregulated in HCC tissues (**Figure 4B**, P <0.05). To further validate the differences in protein expression of the six genes, representative immunohistochemical images from the HPA database were obtained. G6PD, AKR1B15, HMMR, and CSPG5 expression in HCC tissues was significantly higher than in the adjacent normal liver tissues (**Figure 4C**). However, the expression of ELOVL 3 and FABP 6 was negative in both HCC and adjacent normal liver tissues (**Figure S1**).

### Correlation Analysis Between Clinicopathological Characteristics and the Metabolism-Related Gene Signature

**Figure 5:**
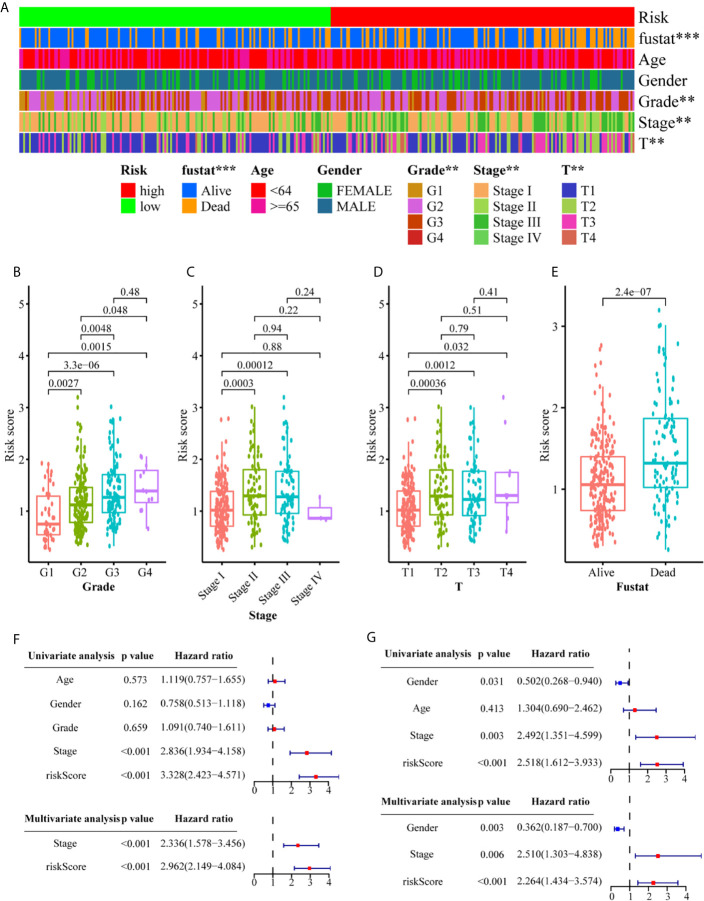
Associations between the signature risk scores and clinicopathological characteristics. **(A)** Heatmap of the clinicopathological characteristics and the risk score; **(B–E)** The risk score in different groups divided by clinical characteristics. Univariate Cox regression analyses and multivariate Cox regression analyses regarding OS in the TCGA **(F)** and the ICGC **(G)** set. **P<0.01; ***P<0.001.

We conducted a series of correlation analyses to study the relationship between the metabolism-related gene signature and clinicopathological characteristics. The results of the heat map (**Figure 5A**) and scatter diagrams (**Figures 5B–E**) showed that tumor grade, clinical stage, T stage, and survival status were significantly related to the risk score. Next, we conducted univariate and multivariate Cox analyses to evaluate and validate whether the gene signature represents an independent risk factor for OS (**Figures 5F, G**). Univariate Cox regression analysis indicated that the risk score (training set: HR = 3.328, 95% CI = 2.423–4.571, *P <*0.001; validation set, HR = 2.518, 95% CI = 1.612–3.933, *P <*0.001) and tumor stage (training set: HR = 2.836, 95% CI = 1.934–4.158, *P <*0.001; validation set, HR = 2.492, 95% CI = 1.351–4.599, *P <*0.01) were closely correlated with OS. Multivariate Cox regression analysis further confirmed that the risk score was an independent risk predictor of OS (training set: HR = 2.962, 95% CI = 2.149–4.084, *P <*0.001; validation set: HR = 2.264, 95% CI = 1.434–3.574, *P <*0.001). These results indicate that the six-gene signature is an independent risk indicator for the prognostic evaluation of HCC.

### Construction and Validation of a Predictive Nomogram

**Figure 6:**
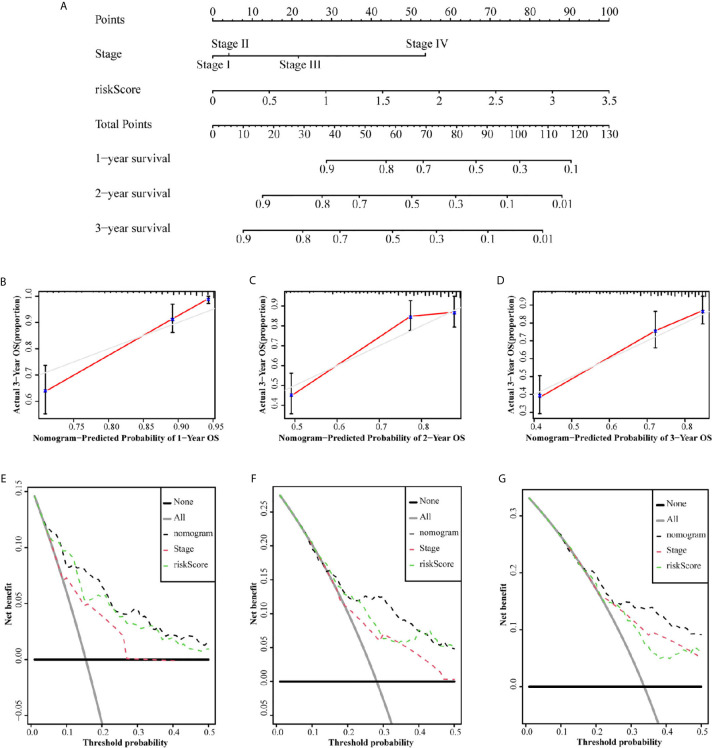
Nomograms to predict OS in hepatocellular carcinoma patients. **(A)** Nomograms using clinical traits shared between the TCGA set. The calibration and DCA curve for determining the reliability of the nomogram to predict the 1-year **(B, E)**, 2-year **(C, F)** and 3-year **(D, G)** OS.

Since independent prognostic analysis confirmed that tumor stage and risk score were independent risk factors for HCC, we constructed a nomogram to estimate the probability of 1-, 2-, and 3-year OS (**Figure 6A**). The calibration chart showed that the OS probability predicted by the nomogram approximated the actual OS probability well, especially for the 3-year OS probability (**Figures 6B–D**). Moreover, the C-index of the nomogram (0.735, 95% CI: 0.710–0.760) was higher than that of both stage (0.637, 95% CI: 0.609–0.665) and risk score (0.716, 95% CI: 0.692–0.741). We further constructed a DCA curve to predict the reliability of the nomogram (**Figures 6E–G**). The results confirmed that the nomogram provides the highest reliability, in comparison to single tumor stage and risk score, especially for predicting 3-year OS.

### Functional Analyses

**Figure 7:**
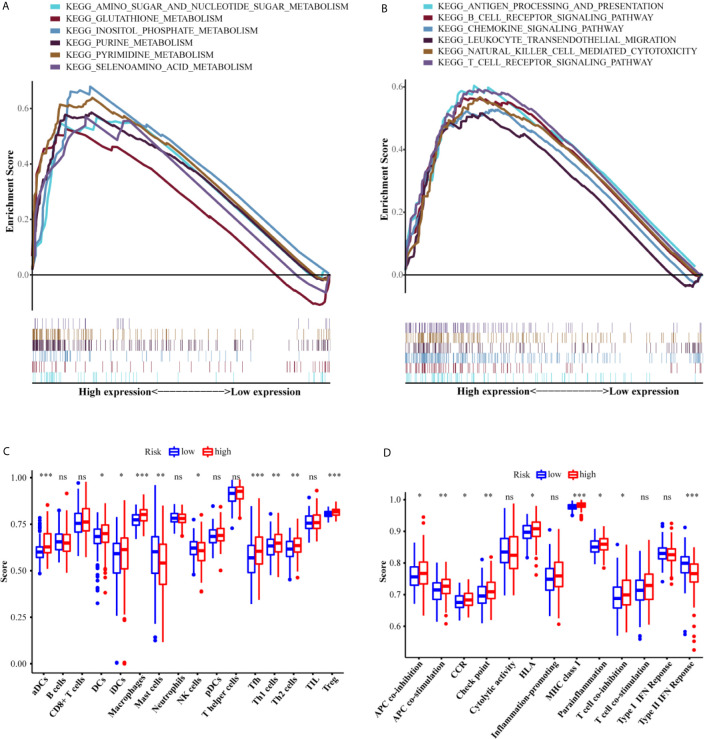
Functional analyses of the low-risk and high-risk groups. The differences of the metabolism-related **(A)** and immune-related **(B)** pathways between high-risk and low-risk groups. The scores of 16 immune cells **(C)** and 13 immune-related functions **(D)** between the two risk groups. Adjusted *P* values were showed as: ns, not significant; *P <0.05; **P <0.01; ***P <0.001.

To elucidate the molecular mechanism associated with the six-gene signature, GSEA was applied to the training set. As we have seen, the metabolism-related KEGG pathways were significantly enriched in the high-risk group (**Figures 7A, B**). These KEGG pathways include amino sugar and nucleotide sugar metabolism (FDR = 0.013, *P* = 0.002), glutathione metabolism (FDR = 0.311, *P* = 0.024), inositol phosphate metabolism (FDR = 0.003, *P <*0.000), purine metabolism (FDR = 0.002, *P <*0.000), pyrimidine metabolism (FDR = 0.002, *P <*0.000), and selenoamino acid metabolism (FDR = 0.028, *P* = 0.004). Interestingly, many immune-related responses in the high-risk group were also significantly enriched, including antigen processing and presentation (FDR = 0.032, *P* = 0.040), B cell receptor signaling pathway (FDR = 0.040, *P* = 0.026), chemokine signaling pathway (FDR = 0.029, *P* = 0.016), leukocyte transendothelial migration (FDR = 0.038, *P* = 0.018), natural killer cell mediated cytotoxicity (FDR = 0.017, *P* = 0.010), and T cell receptor signaling pathway (FDR = 0.027, *P* = 0.010).

An increasing number of studies have indicated that metabolic reprogramming of tumors is related to immune infiltration and immune responses (16). Therefore, we further investigated whether there were different subgroups of immune cells in the two risk groups and scored the related functions by using ssGSEA. The scores of the immune cells (including aDCs, DCs, iDCs, macrophages, mast cells, NK cells, Tfh cells, Th1 cells, Th2 cells, and Treg cells) were significantly different between the two risk groups (*P <*0.05, **Figure 7C**). Additionally, the high-risk group showed higher scores for APC_co_stimulation, APC_co_inhibition, CCR, Check-point, HLA, MHC_class_I, Parainflammation, and T_cell_co-inhibition, while the score for Type_II_IFN_Response exhibited the opposite trend (*P <*0.05). The ICGC set study confirmed the differences between the two risk groups in aDC cells, DC cells, iDC cells, macrophages cells, NK cells, TH2 cells, Treg cells, APC_co_stimulation, check-point, and HLA (*P <*0.05, **Figure 7D**).

### Relationship Between Metabolism-Related Gene Signature and Expression of Immune Checkpoints

**Figure 8:**
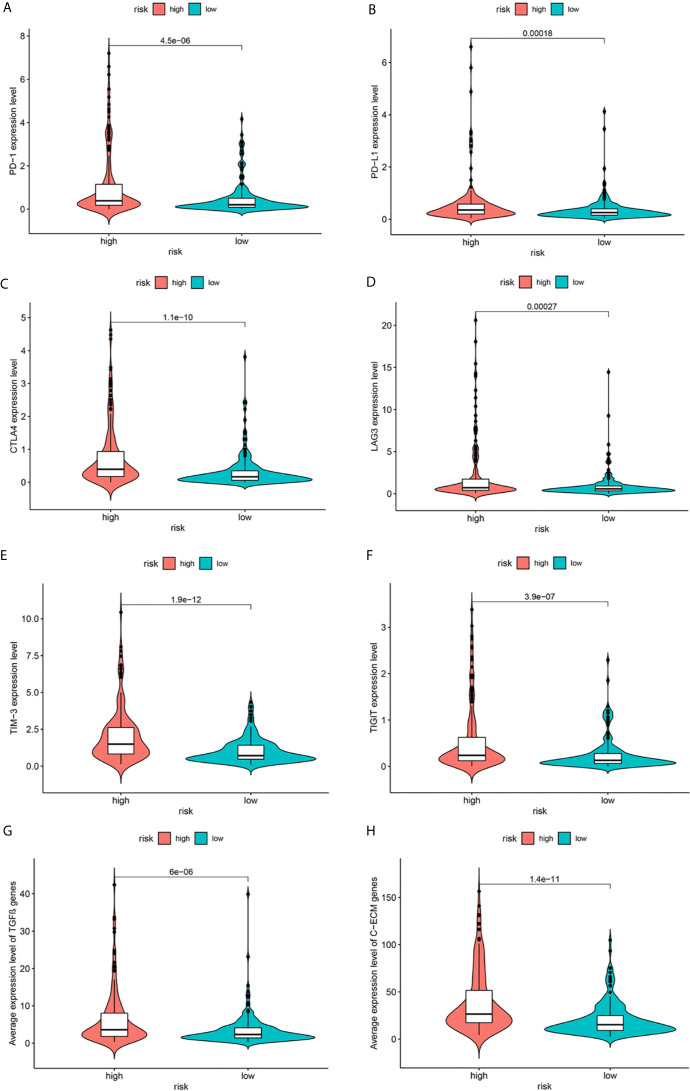
Expression of immune checkpoints **(A–F)**, TGF-β–encoding **(G)** and C-ECM **(H)** signature genes.

Immune-based therapies have become a systematic treatment method for improving the prognosis of advanced cancers (17). Therefore, we further analyzed the correlation between the risk groups and the expression levels of immune checkpoint proteins. As shown in **Figures 8A–F**, in comparison with the low-risk group, the high-risk group had significantly higher expression levels of programmed death-1 (PD-1), programmed death ligand 1 (PD-L1), cytotoxic T-lymphocyte-associated protein 4 (CTLA-4), lymphocyte activation gene-3 (LAG3), T-cell immunoglobulin and mucin-domain containing-3 (TIM-3), and T cell immunoreceptor with Ig and ITIM domains (TIGIT). Moreover, recent studies have indicated that TGF-β signaling and a cohort of 30 extracellular matrix genes (C-ECM) are significantly associated with cancer immunosuppression and poor prognosis. **Figures 8G, H** describe the increased expression of these genes in the high-risk group.

## Discussion

HCC has become a major health concern and threat to global mortality, especially in China. From 1991 to 2011, the mortality rate associated with liver cancer has continuously increased (18). According to statistics, in 2015, there were approximately 370,000 new cases of liver cancer, and 326,000 related deaths (19). Progress in radical surgery plays a vital role in alleviating the global burden of HCC. However, the long-term outcome of HCC remains unclear. Therefore, it is essential to establish an effective prognostic signature for the evaluation and treatment of HCC.

In this study, we used the transcriptome profiling and the corresponding clinical information of HCC patients, obtained from the TCGA database, to identify a six metabolism-related gene signature (G6PD, AKR1B15, HMMR, CSPG5, ELOVL3, FABP6) for the prognostic evaluation of HCC, and verified them in the ICGC database. G6PD is a rate-limiting enzyme that catalyzes the pentose phosphate pathway and participates in regulating the redox homeostasis of cells exposed to oxidative damage (20). Abnormal behavior of G6PD is related to a variety of pathological processes and diseases, including inflammation (21), diabetes (22), and tumors (23). High levels of G6PD expression were observed in human livers infected with HBV and HBV-related cancers, which provides further confirmation of the findings presented here (24). AKR1B15, a newly discovered Aldo-keto reductase (AKR), shares 92% amino acid sequence identity with AKR1B10. Studies have shown that AKR1B10 can induce a variety of cancers, such as liver cancer (25), non-small cell lung cancer (26), and pancreatic cancer (27), and represents a promising potential cancer target. Presently, a few studies have indicated that mutation of the AKR1B15 allele is related to mitochondrial oxidative phosphorylation and serous ovarian cancer, which has attracted increasing attention from researchers (28, 29). HMMR is a protein that regulates cell growth and is involved in maintaining homeostasis and the directional regulation of mitotic and meiotic spindles (30). It has been shown that upregulation of HMMR expression is related to the aggressive growth and low survival rate of various cancers, such as breast cancer (31), colorectal cancer (32), and gastric cancer (33). Several other types of cancer, such as breast cancer (34) and malignant peripheral nerve sheath tumors (35), have been found to exhibit poor patient survival, which has been correlated with low expression of HMMR. ELOVL3 is an ultra-long-chain fatty acid elongase that participates in the synthesis of fatty acids (36). A small number of studies have shown that ELOVL3 is highly expressed in colorectal and prostate cancers (37, 38). FABP6 is a protein involved in bile acid digestion, absorption, metabolism and enterohepatic circulation (39). Recent studies have indicated that elevated levels of FABP6 are associated with the initiation and development of colorectal cancer by regulating the NF-κB pathway (40). The relationship between these six genes and the progression of HCC in patients remains to be clarified, as very few studies have examined these genes. In this study, these six genes were all found to be upregulated in HCC tissues and were significantly correlated with a short OS (*P <*0.001).

Furthermore, according to the risk scores calculated based on the expression levels and regression coefficient values of the six metabolism-related genes, we divided the patients into two risk groups. The low-risk group exhibited better OS than the high-risk group. Thus, the six-gene signature also represents an independent prognostic risk indicator for HCC. Based on multivariate Cox analysis, we proposed a nomogram based on tumor stage and risk score. The C-index and DCA curve proved that the nomogram was superior to both tumor stage and single risk score in predicting tumor prognosis, especially for predicting OS in 3 years.

In addition, metabolism-related pathways were identified in the high-risk group. Interestingly, patients in the high-risk group were also more closely associated with changes in immune-related pathways and upregulated expression of immune checkpoint proteins (PD-1, PD-L1, TIM3, TIGIT), TGF-β, and C-ECM genes. Immune checkpoint blocking therapy is promising in the treatment of HCC (41). It has been suggested that HCC patients with higher expression of PD-1 or PD-L1 might be more likely to respond to immune checkpoint blocking therapy (42). Therefore, maybe we could identify high-risk patients early based on our gene signature and conduct immune checkpoint blocking treatment or other applicable therapy to improve the prognosis of patients, which needs further verification in the future. Previous researches have indicated that an increase in tumor-related macrophages (43), NK cells (44), and Treg cells (45) in the tumor microenvironment is related to reduced OS in HCC patients, and strategies for immunotherapy have been proposed. However, it is worth noting that the relationship between metabolism-related genes and immunity remains to be clarified.

This study has several limitations. Our risk signature significantly stratifies HCC patients, which makes the prognosis and immunotherapy response evaluation accurate and reliable. However, our research is mainly based on a public database, it is retrospective, and belongs to a small sample study. Additional studies using large-scale, prospective, and multicenter clinical trials are needed to validate the robustness and reproducibility of the gene signature. Although we have performed a comprehensive bioinformatics analysis to construct and verify the prognostic metabolism-related gene signature in HCC, it may not be as accurate for different types of HCC. Furthermore, the underlying molecular mechanism of how these six genes affect the development of HCC remains unclear, and further experiments are needed. The correlation between risk score and immune status has not been verified by basic experiments, which is an important issue worthy of further study.

## Conclusion

In conclusion, this study developed a prognostic signature comprised of six metabolism-related genes for HCC. The signature was confirmed to be an independent risk indicator related to the OS of HCC in the TCGA and ICGC databases. Moreover, we constructed a nomogram composed of risk score and tumor grade, which provided higher accuracy in predicting the OS of patients with HCC. However, longitudinal clinical experiments should be conducted to verify this hypothesis.

## Data Availability Statement

The original contributions presented in the study are included in the article/**Supplementary Material**. Further inquiries can be directed to the corresponding authors.

## Author Contributions

CY and MY conceived the study, performed the bioinformatics analyses and wrote the manuscript. MC and JO performed the experiments. WT and FD downloaded and organized the clinical and gene expression data. DY, SL and YZ performed the statistical analyses. CZ and YC critically revised the article for essential intellectual content and administrative support. All authors contributed to the article and approved the submitted version.

## Funding

This work was supported by the National Nature Science Foundation of China (82071655, 81860276), Scientific Research Project Foundation of Hubei Provincial Health Commission (WJ2019M179).

## Conflict of Interest

The authors declare that the research was conducted in the absence of any commercial or financial relationships that could be construed as a potential conflict of interest.
