# Supplementary material for: Prognostic Implication of a Novel Metabolism-Related Gene Signature in Hepatocellular Carcinoma
Source: Front Oncol. 2021 Jun 4;11:666199. doi: 10.3389/fonc.2021.666199 (PMC8213025; doi:10.3389/fonc.2021.666199)
Supplement: Supplementary file 1 [file DataSheet_1.docx]

**Prognostic Implication of a Novel Metabolism-Related Gene Signature in** **Hepatocellular Carcinoma**

Chaoyan Yuan^1^†, Mengqin Yuan^2^†, Mingqian Chen^1^, Jinhua Ouyang^1^, Wei Tan^2^, Fangfang Dai^2^, Dongyong Yang^2^, Shiyi Liu^2^, Yajing Zheng^2^, Chenliang Zhou^3*^, Yanxiang Cheng^2*^

**Supplementary Figures and Tables**

**Supplementary Figures**

**Figure S1.** The immunohistochemistry staining images of ELOVL3 and FABP6 from the HPA.

**Supplementary Tables**

**Table S1.** The primer sequences of the eight prognostic genes.

**Table S2.** List of metabolic related genes collected from MSigDB.

**Table S3.** The results of differential expression analysis.

**Table S4.** The results of univariate COX regression analysis.


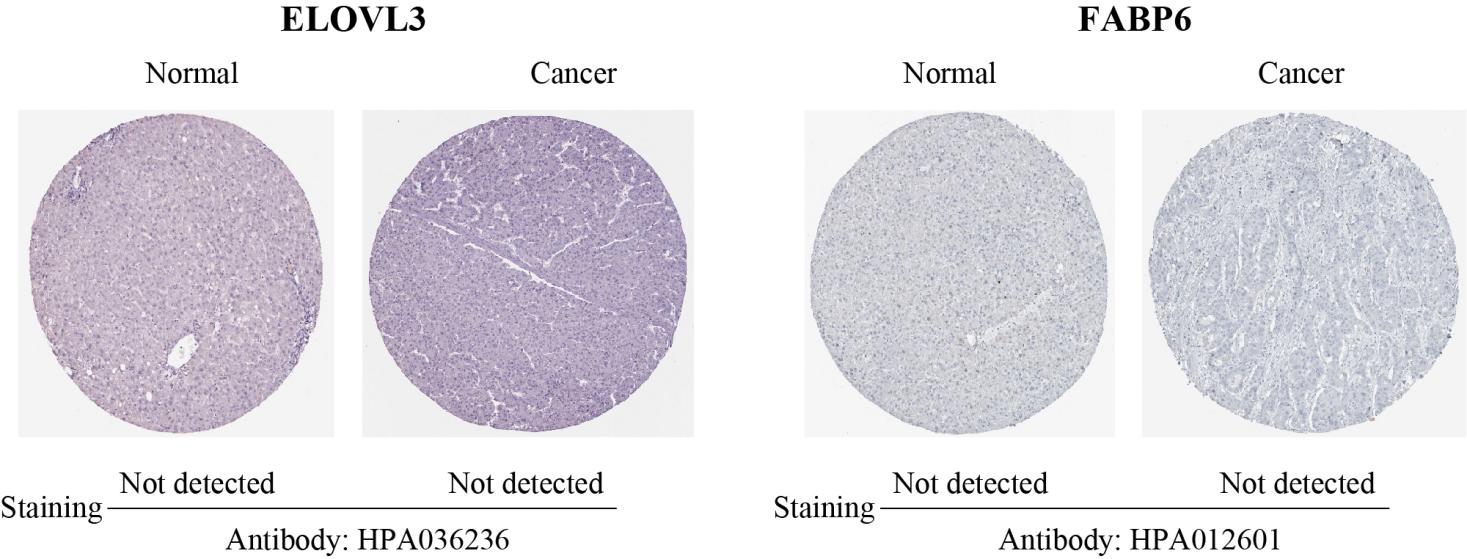


**Figure S1.** The immunohistochemistry staining images of ELOVL3 and FABP6 from the HPA.

**Table S1. The primer sequences of the eight prognostic genes.**

| Gene | Forward Primer | Reverse Primer |
| --- | --- | --- |
| G6PD | 5'‐AACATCGCCTGCGTTATCCT‐3' | 5'‐GGTGGTTCTGCATCACGTCC‐3' |
| AKR1B15 | 5'‐GTCAGCAGCTATTGGCACGAC‐3' | 5'‐CACTTTGCCGAGAAGAGATGC‐3' |
| HMMR | 5'‐ATGGTGCAGCTCAGGAACAG‐3' | 5'‐GATACTTCCGATTTGAGTTGGC‐3' |
| CSPG5 | 5'‐TCTGGCATGAGGAGTTTACCAG‐3' | 5'‐ACGGTCCCATTGCTGTCTTAG‐3' |
| ELOVL3 | 5'‐TGCCAGACCTACATCAGGCC‐3' | 5'‐TCATTCTCCCAAAACTAAGCCC‐3' |
| FABP6 | 5'‐ATGGGCAGGACTTCACTTGG‐3' | 5'‐TCATAGGTCACGCCTCCGAT‐3' |
| ACTB | 5'‐GTCCACCGCAAATGCTTCTA‐3' | 5'‐TGCTGTCACCTTCACCGTTC‐3' |

**Table S2. List of metabolic related genes collected from MSigDB.**

As shown in separate Table S2.xlsx.

**Table S3. The results of differential expression analysis.**

| **gene** | **Normal_Mean** | **Tumor_Mean** | **logFC** | ***P* Value** | **fdr** |
| --- | --- | --- | --- | --- | --- |
| PYCR1 | 0.8616 | 8.1212 | 3.2367 | 0.0000 | 0.0000 |
| B3GALT1 | 0.0037 | 0.1311 | 5.1496 | 0.0002 | 0.0002 |
| CPNE7 | 0.1175 | 1.1621 | 3.3063 | 0.0000 | 0.0000 |
| B4GALNT2 | 0.0021 | 0.5520 | 8.0396 | 0.0000 | 0.0000 |
| GPC3 | 2.9286 | 271.5420 | 6.5348 | 0.0000 | 0.0000 |
| BCAN | 0.0196 | 0.7117 | 5.1822 | 0.0000 | 0.0000 |
| TYRP1 | 0.0086 | 0.2677 | 4.9602 | 0.0004 | 0.0005 |
| G6PD | 1.3117 | 13.5821 | 3.3722 | 0.0000 | 0.0000 |
| SLC5A5 | 0.0070 | 0.0672 | 3.2706 | 0.0001 | 0.0001 |
| FABP4 | 0.9997 | 9.5899 | 3.2620 | 0.0000 | 0.0000 |
| CYP17A1 | 0.7641 | 22.7908 | 4.8986 | 0.0000 | 0.0000 |
| ACAN | 0.0087 | 0.1922 | 4.4653 | 0.0000 | 0.0000 |
| AKR1B15 | 0.0990 | 2.0014 | 4.3373 | 0.0000 | 0.0000 |
| HMMR | 0.1560 | 2.4914 | 3.9970 | 0.0000 | 0.0000 |
| SLC6A7 | 0.0037 | 0.0498 | 3.7487 | 0.0000 | 0.0000 |
| ALOX15B | 0.1421 | 1.8179 | 3.6776 | 0.0000 | 0.0000 |
| CERS1 | 0.0071 | 0.2480 | 5.1223 | 0.0000 | 0.0000 |
| B4GALNT1 | 0.0348 | 1.0366 | 4.8971 | 0.0000 | 0.0000 |
| HPSE2 | 0.0025 | 0.0365 | 3.8746 | 0.0000 | 0.0000 |
| SLC6A8 | 0.4970 | 6.2503 | 3.6527 | 0.0000 | 0.0000 |
| CYP2C19 | 3.0853 | 0.3821 | -3.0132 | 0.0000 | 0.0000 |
| CSPG5 | 0.0392 | 0.3718 | 3.2464 | 0.0000 | 0.0000 |
| STAB2 | 2.5142 | 0.1269 | -4.3085 | 0.0000 | 0.0000 |
| DUOX1 | 0.0569 | 0.4941 | 3.1175 | 0.0000 | 0.0000 |
| SLC44A5 | 0.0988 | 0.8601 | 3.1215 | 0.0057 | 0.0068 |
| ALPI | 0.0089 | 4.6185 | 9.0176 | 0.0000 | 0.0000 |
| ELOVL3 | 0.0288 | 0.2640 | 3.1986 | 0.0000 | 0.0000 |
| RPL10L | 0.0063 | 0.4602 | 6.1857 | 0.0000 | 0.0000 |
| PLA2G4F | 0.0041 | 0.0708 | 4.1122 | 0.0002 | 0.0003 |
| NAT8L | 0.0206 | 0.4011 | 4.2797 | 0.0000 | 0.0000 |
| CEMIP | 0.0875 | 0.9283 | 3.4065 | 0.0000 | 0.0000 |
| CKMT1A | 0.0069 | 0.1345 | 4.2876 | 0.0000 | 0.0000 |
| NQO1 | 1.4597 | 57.9538 | 5.3112 | 0.0000 | 0.0000 |
| CHST6 | 0.0033 | 0.0349 | 3.4124 | 0.0000 | 0.0000 |
| FABP6 | 0.0104 | 0.4431 | 5.4166 | 0.0000 | 0.0000 |
| CYP19A1 | 0.0019 | 0.3541 | 7.5225 | 0.0000 | 0.0000 |
| FUT2 | 0.0684 | 0.8833 | 3.6912 | 0.0000 | 0.0000 |
| CKMT1B | 0.0071 | 0.2947 | 5.3661 | 0.0042 | 0.0051 |
| DIO2 | 0.0154 | 0.4798 | 4.9655 | 0.0000 | 0.0000 |
| NDST3 | 0.1681 | 0.0123 | -3.7719 | 0.0000 | 0.0000 |

**Table S4. The results of univariate COX regression analysis.**

| **gene** | **HR** | **HR.95L** | **HR.95H** | ***P* value** |
| --- | --- | --- | --- | --- |
| PSMD9 | 2.3421 | 1.5028 | 3.6501 | 0.0002 |
| NUP107 | 2.0685 | 1.4933 | 2.8654 | 0.0000 |
| PIP4K2A | 1.5938 | 1.2219 | 2.0789 | 0.0006 |
| MED19 | 2.5965 | 1.7760 | 3.7959 | 0.0000 |
| GNPDA1 | 1.7286 | 1.3766 | 2.1707 | 0.0000 |
| GDPD1 | 1.8802 | 1.3536 | 2.6116 | 0.0002 |
| PGD | 1.5780 | 1.3052 | 1.9078 | 0.0000 |
| PPARGC1A | 0.7452 | 0.6357 | 0.8734 | 0.0003 |
| MBOAT2 | 1.8062 | 1.2863 | 2.5361 | 0.0006 |
| MED6 | 2.6046 | 1.6456 | 4.1226 | 0.0000 |
| PTGS1 | 1.5188 | 1.2088 | 1.9082 | 0.0003 |
| NUP210 | 1.4750 | 1.1897 | 1.8289 | 0.0004 |
| SLC10A1 | 0.8719 | 0.8125 | 0.9357 | 0.0001 |
| NUP62 | 1.8233 | 1.3772 | 2.4139 | 0.0000 |
| RARS1 | 2.3650 | 1.5779 | 3.5447 | 0.0000 |
| PSME3 | 1.8113 | 1.3209 | 2.4837 | 0.0002 |
| AMD1 | 2.1909 | 1.6211 | 2.9610 | 0.0000 |
| SEC13 | 1.8135 | 1.2799 | 2.5695 | 0.0008 |
| GAPDH | 1.5362 | 1.2358 | 1.9096 | 0.0001 |
| PSMA5 | 1.7146 | 1.2506 | 2.3507 | 0.0008 |
| GNPDA2 | 2.2404 | 1.3961 | 3.5953 | 0.0008 |
| ABCC5 | 2.4302 | 1.6757 | 3.5244 | 0.0000 |
| NUP93 | 2.1038 | 1.4896 | 2.9713 | 0.0000 |
| B3GALNT1 | 1.7239 | 1.2998 | 2.2864 | 0.0002 |
| DLAT | 1.6778 | 1.2976 | 2.1696 | 0.0001 |
| CYP2C9 | 0.8546 | 0.7955 | 0.9180 | 0.0000 |
| ACAT1 | 0.6661 | 0.5492 | 0.8079 | 0.0000 |
| FABP5 | 1.3857 | 1.1582 | 1.6579 | 0.0004 |
| SLC44A1 | 1.7944 | 1.2861 | 2.5036 | 0.0006 |
| GPC1 | 1.3406 | 1.1489 | 1.5643 | 0.0002 |
| ME1 | 1.2454 | 1.1000 | 1.4101 | 0.0005 |
| PKM | 1.2298 | 1.1088 | 1.3640 | 0.0001 |
| B3GAT3 | 1.7090 | 1.3249 | 2.2045 | 0.0000 |
| AGRN | 1.3692 | 1.1723 | 1.5991 | 0.0001 |
| TXNRD1 | 1.3572 | 1.1598 | 1.5882 | 0.0001 |
| NPAS2 | 1.6866 | 1.2857 | 2.2125 | 0.0002 |
| PSMB2 | 2.0801 | 1.4889 | 2.9060 | 0.0000 |
| GLTP | 2.0601 | 1.4458 | 2.9354 | 0.0001 |
| GSR | 1.5756 | 1.2861 | 1.9303 | 0.0000 |
| PFKFB4 | 2.0087 | 1.5835 | 2.5480 | 0.0000 |
| NUP37 | 1.9875 | 1.4277 | 2.7668 | 0.0000 |
| PRXL2B | 1.5734 | 1.2744 | 1.9426 | 0.0000 |
| IVD | 0.6195 | 0.4809 | 0.7981 | 0.0002 |
| PLEKHA8 | 3.1408 | 1.8148 | 5.4357 | 0.0000 |
| MBOAT7 | 1.9395 | 1.4599 | 2.5767 | 0.0000 |
| TPI1 | 1.6058 | 1.2457 | 2.0699 | 0.0003 |
| KPNB1 | 1.7151 | 1.3004 | 2.2622 | 0.0001 |
| ASNS | 1.3499 | 1.1466 | 1.5893 | 0.0003 |
| ACOT7 | 1.5562 | 1.2214 | 1.9829 | 0.0003 |
| CHPF2 | 1.7762 | 1.3069 | 2.4140 | 0.0002 |
| G6PD | 1.4435 | 1.2858 | 1.6205 | 0.0000 |
| B4GALT5 | 1.5256 | 1.2439 | 1.8711 | 0.0000 |
| PSMD13 | 1.7665 | 1.2784 | 2.4409 | 0.0006 |
| HEXB | 1.7310 | 1.2568 | 2.3840 | 0.0008 |
| PSMA1 | 2.5550 | 1.7006 | 3.8388 | 0.0000 |
| SEH1L | 2.2648 | 1.4215 | 3.6082 | 0.0006 |
| ENO1 | 1.6763 | 1.3953 | 2.0139 | 0.0000 |
| DGAT2L6 | 20.1285 | 3.5183 | 115.1551 | 0.0007 |
| MED17 | 2.3316 | 1.4231 | 3.8200 | 0.0008 |
| TKT | 1.3334 | 1.1496 | 1.5466 | 0.0001 |
| FTCD | 0.8435 | 0.7757 | 0.9172 | 0.0001 |
| CTSA | 1.6902 | 1.2980 | 2.2010 | 0.0001 |
| AKR1B15 | 1.2973 | 1.1316 | 1.4874 | 0.0002 |
| PHKA2 | 1.7970 | 1.3496 | 2.3928 | 0.0001 |
| RAN | 2.0447 | 1.5645 | 2.6724 | 0.0000 |
| HS2ST1 | 1.8272 | 1.3212 | 2.5271 | 0.0003 |
| NUP188 | 1.6947 | 1.2678 | 2.2653 | 0.0004 |
| HMMR | 1.6792 | 1.3780 | 2.0462 | 0.0000 |
| SARS1 | 1.7606 | 1.3182 | 2.3513 | 0.0001 |
| GOT2 | 0.6143 | 0.4911 | 0.7685 | 0.0000 |
| CERS5 | 1.8541 | 1.3154 | 2.6136 | 0.0004 |
| PFKP | 1.2505 | 1.1144 | 1.4032 | 0.0001 |
| ELOVL1 | 2.0623 | 1.5535 | 2.7378 | 0.0000 |
| NFYC | 1.9053 | 1.3046 | 2.7824 | 0.0008 |
| LALBA | 1513.3530 | 29.9927 | 76359.8157 | 0.0003 |
| GCDH | 0.6825 | 0.5462 | 0.8530 | 0.0008 |
| NUP85 | 2.0610 | 1.5210 | 2.7927 | 0.0000 |
| ACOT12 | 0.8043 | 0.7148 | 0.9051 | 0.0003 |
| ABCC1 | 1.4192 | 1.1916 | 1.6903 | 0.0001 |
| GYG1 | 1.9139 | 1.4178 | 2.5834 | 0.0000 |
| PSMD1 | 3.2071 | 2.1117 | 4.8706 | 0.0000 |
| GAPDHS | 13675175.8277 | 1064.1244 | 175741145674.2050 | 0.0007 |
| CSPG5 | 2.0167 | 1.4011 | 2.9027 | 0.0002 |
| IYD | 0.6912 | 0.5548 | 0.8610 | 0.0010 |
| PPP1CB | 2.0204 | 1.4707 | 2.7754 | 0.0000 |
| RPE | 2.0602 | 1.4389 | 2.9498 | 0.0001 |
| PSMD14 | 2.3337 | 1.6889 | 3.2248 | 0.0000 |
| PSMD11 | 1.9374 | 1.3676 | 2.7445 | 0.0002 |
| PON1 | 0.8427 | 0.7808 | 0.9096 | 0.0000 |
| SGPP2 | 1.3205 | 1.1341 | 1.5376 | 0.0003 |
| B4GALT3 | 1.7961 | 1.3224 | 2.4396 | 0.0002 |
| PTPMT1 | 2.1191 | 1.4462 | 3.1053 | 0.0001 |
| CSAD | 0.6840 | 0.5648 | 0.8284 | 0.0001 |
| STARD5 | 0.3993 | 0.2485 | 0.6416 | 0.0001 |
| B4GALT2 | 1.8278 | 1.3811 | 2.4189 | 0.0000 |
| SMOX | 1.4790 | 1.2499 | 1.7501 | 0.0000 |
| GLS | 1.3624 | 1.1332 | 1.6380 | 0.0010 |
| SRM | 1.5740 | 1.2654 | 1.9578 | 0.0000 |
| MED8 | 3.0644 | 2.1137 | 4.4427 | 0.0000 |
| SUMO2 | 1.7357 | 1.2591 | 2.3925 | 0.0008 |
| CPS1 | 0.8931 | 0.8386 | 0.9511 | 0.0004 |
| MTMR2 | 2.0976 | 1.5863 | 2.7736 | 0.0000 |
| MED10 | 1.9153 | 1.4679 | 2.4991 | 0.0000 |
| GPD1L | 1.5431 | 1.2372 | 1.9244 | 0.0001 |
| SLC27A5 | 0.8386 | 0.7639 | 0.9205 | 0.0002 |
| SMS | 2.1140 | 1.6395 | 2.7258 | 0.0000 |
| PPT1 | 1.6862 | 1.3470 | 2.1108 | 0.0000 |
| ASRGL1 | 1.3637 | 1.1490 | 1.6185 | 0.0004 |
| NUP43 | 2.1651 | 1.5481 | 3.0280 | 0.0000 |
| ENOPH1 | 1.6957 | 1.2526 | 2.2954 | 0.0006 |
| GALNS | 1.7312 | 1.3022 | 2.3016 | 0.0002 |
| AGPS | 1.9518 | 1.4149 | 2.6926 | 0.0000 |
| LPCAT4 | 1.5145 | 1.2186 | 1.8823 | 0.0002 |
| MED15 | 1.8207 | 1.3096 | 2.5311 | 0.0004 |
| SLC2A1 | 1.5805 | 1.3499 | 1.8505 | 0.0000 |
| CSNK2A2 | 2.1012 | 1.3947 | 3.1656 | 0.0004 |
| ELOVL3 | 1.8141 | 1.3608 | 2.4183 | 0.0000 |
| SORD | 0.7947 | 0.6970 | 0.9061 | 0.0006 |
| ODC1 | 1.3658 | 1.1472 | 1.6261 | 0.0005 |
| CYP27A1 | 0.8163 | 0.7290 | 0.9141 | 0.0004 |
| STARD3NL | 1.8436 | 1.3762 | 2.4697 | 0.0000 |
| HAO1 | 0.8527 | 0.7790 | 0.9333 | 0.0005 |
| BCAT1 | 1.5977 | 1.2742 | 2.0033 | 0.0000 |
| G6PC | 0.8525 | 0.7886 | 0.9216 | 0.0001 |
| RIMKLA | 3.0143 | 1.6552 | 5.4893 | 0.0003 |
| HK2 | 1.3106 | 1.1248 | 1.5270 | 0.0005 |
| TALDO1 | 1.4579 | 1.1834 | 1.7961 | 0.0004 |
| AACS | 1.7917 | 1.3213 | 2.4296 | 0.0002 |
| MARS1 | 2.1838 | 1.5749 | 3.0283 | 0.0000 |
| RPIA | 1.9558 | 1.4324 | 2.6705 | 0.0000 |
| MED22 | 1.9961 | 1.4519 | 2.7444 | 0.0000 |
| PPP1CC | 1.8760 | 1.3671 | 2.5743 | 0.0001 |
| TBL1XR1 | 2.1603 | 1.5543 | 3.0025 | 0.0000 |
| BDH1 | 0.7369 | 0.6385 | 0.8504 | 0.0000 |
| ALDOA | 1.3654 | 1.1823 | 1.5769 | 0.0000 |
| PSMD2 | 1.9742 | 1.4547 | 2.6793 | 0.0000 |
| PSPH | 1.3589 | 1.1357 | 1.6260 | 0.0008 |
| RAE1 | 2.3319 | 1.6222 | 3.3521 | 0.0000 |
| CERS6 | 1.6654 | 1.2739 | 2.1771 | 0.0002 |
| SRD5A3 | 1.5780 | 1.2066 | 2.0637 | 0.0009 |
| PSMA7 | 1.6356 | 1.2260 | 2.1820 | 0.0008 |
| FABP6 | 1.5304 | 1.2588 | 1.8604 | 0.0000 |
| ACACA | 1.6746 | 1.2654 | 2.2160 | 0.0003 |
| GPD2 | 1.9576 | 1.4629 | 2.6196 | 0.0000 |
| NUP155 | 2.2356 | 1.6142 | 3.0962 | 0.0000 |
| GLA | 1.4224 | 1.1566 | 1.7493 | 0.0008 |
| PSMD6 | 2.7647 | 1.6273 | 4.6972 | 0.0002 |
| PLBD1 | 1.3794 | 1.2003 | 1.5852 | 0.0000 |
| HILPDA | 1.4769 | 1.2752 | 1.7106 | 0.0000 |
| NDC1 | 2.3004 | 1.7208 | 3.0752 | 0.0000 |
| B4GALT4 | 1.7990 | 1.2709 | 2.5465 | 0.0009 |
| DMGDH | 0.7934 | 0.6986 | 0.9011 | 0.0004 |
| LCLAT1 | 2.3280 | 1.5819 | 3.4262 | 0.0000 |
| RPL17 | 1.5769 | 1.2166 | 2.0438 | 0.0006 |
| LHB | 2.1877 | 1.4329 | 3.3402 | 0.0003 |
| EEF1E1 | 1.7294 | 1.3500 | 2.2155 | 0.0000 |
| IARS1 | 1.8142 | 1.3968 | 2.3563 | 0.0000 |
| LARS1 | 2.0354 | 1.4239 | 2.9094 | 0.0001 |
| NUP205 | 2.0585 | 1.5247 | 2.7793 | 0.0000 |
| PLAAT1 | 2.7553 | 1.5980 | 4.7507 | 0.0003 |
| LPCAT1 | 1.5373 | 1.3263 | 1.7818 | 0.0000 |
| PTDSS2 | 1.6954 | 1.3439 | 2.1388 | 0.0000 |
